# Effects of tidal volume challenge on the reliability of plethysmography variability index in hepatobiliary and pancreatic surgeries: a prospective interventional study

**DOI:** 10.1007/s10877-023-00977-8

**Published:** 2023-03-18

**Authors:** J. M. Botros, Y. S. M. Salem, M. Khalil, M. F. Algyar, H. M. Yassin

**Affiliations:** 1 Department of Anesthesia and Intensive Care, Fayoum University Hospital, Fayoum University, Fayoum government, Egypt; 2 Department of Anesthesia and Intensive Care, National Liver Institute, Menoufia University, Menoufia government, Egypt; 3 Department of Anesthesiology, Surgical Intensive Care Unit and pain management, Kafrelsheikh University, Kafrelsheikh government, Egypt

**Keywords:** Fluid responsiveness, Low tidal volume, Plethysmography variability index, Tidal volume challenge

## Abstract

**Background:**

The plethysmography variability index (PVI) is a non-invasive, real-time, and automated parameter for evaluating fluid responsiveness, but it does not reliably predict fluid responsiveness during low tidal volume (V_T_) ventilation. We hypothesized that in a ‘tidal volume challenge’ with a transient increase in tidal volume from 6 to 8 ml Kg^− 1^, the changes in PVI could predict fluid responsiveness reliably.

**Method:**

We performed a prospective interventional study in adult patients undergoing hepatobiliary or pancreatic tumor resections and receiving controlled low V_T_ ventilation. The values for PVI, perfusion index, stroke volume variation, and stroke volume index (SVI) were recorded at baseline V_T_ of 6 ml Kg^− 1^, 1 min after the V_T_ challenge (8 ml Kg^− 1^), 1 min after V_T_ 6 ml Kg^− 1^ reduced back again, and then 5 min after crystalloid fluid bolus 6 ml kg^− 1^ (actual body weight) administered over 10 min. The fluid responders were identified by SVI rise ≥ 10% after the fluid bolus.

**Results:**

The area under the receiver operating characteristic curve for PVI value change (ΔPVI_6–8_) after increasing V_T_ from 6 to 8 ml Kg^− 1^ was 0.86 (95% confidence interval, 0.76–0.96), *P* < 0.001, 95% sensitivity, 68% specificity, and with best cut-off value of absolute change (ΔPVI_6–8_) = 2.5%.

**Conclusion:**

In hepatobiliary and pancreatic surgeries, tidal volume challenge improves the reliability of PVI for predicting fluid responsiveness and changes in PVI values obtained after tidal volume challenge are comparable to the changes in SVI.

## Introduction

Hepatobiliary and pancreatic surgeries have been associated with high mortality and morbidity rates, but the latest improvements in anesthesia and surgery management have significantly limited the operative hazards [1]. The management of patients undergoing these major surgeries in the perioperative period is frequently not at ease due to coexisting diseases or debilitation found in many patients as well as due to the potential for significant operative blood loss [2]. The liberal intravenous fluids administration may lead to deleterious pulmonary congestion, impaired wound healing, and acute kidney injury. On the other hand, intraoperative fluid restriction approach targeting a net balance of zero may impair perfusion of vital organs and lead to significantly higher rates of acute kidney injury, oliguria, and renal-replacement therapy [3]. Messina and colleagues reported no significant difference between the two approaches in the incidence of the postoperative major complications or mortality but they reported significant higher incidence of postoperative renal complication in the fluid restriction approach targeting a net balance of zero for elective abdominal surgery in a subgroup analysis [4]. During major hepatobiliary and pancreatic surgeries, intravascular volume expansion is continuously required, but the margin of error is rather narrow [5]. The intraoperative fluid administration is better to be individualized and customed for case by case requirements guided by valid and reliable dynamic index tools. The plethysmography variability index (PVI) is an automated, real-time, and non-invasive dynamic parameter with good potential to predict the fluid responsiveness. PVI estimates the respiratory changes gained by the pulse oximeter waveform [6, 7]. PVI is based on continuous calculations of the respirational alterations of the amplitude of the pulse oximetry waveform of mechanically ventilated patients. These respirational alterations produced by cardiopulmonary interactions, that are markedly affected by lung mechanics determinants as tidal volume (V_T_) values, could significantly influence PVI values [8]. It is presumed that V_T_ ≥ 8 ml kg^− 1^ could significantly alter PVI values for certain patients groups [10]. Using low V_T_ for mechanical ventilation is currently the preferred policy for safe lung protection at intensive care units (ICU) and during anesthesia conducts [10]. The ‘tidal volume challenge’ is proved to be a valid test that help to improve the reliability of dynamic indexes for predicting fluid responsiveness in patients receiving low V_T_ ventilation by transiently increasing V_T_ from 6 to 8 ml kg^− 1^ of predicted body weight (PBW) [11–13]. Up to our best knowledge, there are no studies that evaluated the potential of V_T_ challenge to improve the reliability of PVI in predicting fluid responsiveness in patients receiving low V_T_ ventilation. In this study, we investigated whether a transient increase in V_T_ from 6 to 8 ml kg^− 1^ could improve the predictability of fluid responsiveness by using PVI values change in patients receiving low V_T_ ventilation during hepatobiliary and pancreatic tumor resection surgeries.

## Methods

We performed this prospective interventional study on adult patients undergoing hepatobiliary and pancreatic tumor resection surgeries after obtaining approval from the Ethical and Scientific Committee of Fayoum University Hospital (D154) and the National Liver Institute (0146/2018), Egypt. We received written informed consent from the patients or their surrogates to participate in this study. We registered this study at *Clinicaltrials.gov* (NCT03546179). The study was conducted at the National Liver Institute Hospital, Menoufia, Egypt. We studied patients aged ≥ 18 years undergoing open (non-laparoscopic) hepatobiliary or pancreatic tumor resection. Patients with preoperative cardiac arrhythmias, peripheral vascular disease, low left ventricular function (ejection fraction < 40%), significant valvular heart disease, metastasis, irresectable tumor, massive blood loss, or fulminant hepatic failure were excluded from this study.

On the day of surgery, intravenous access was obtained. Demographic and anthropometric data including age, sex, height, actual body weight (ABW), PBW, body mass index (BMI), body surface area (BSA), smoking history, comorbid diseases, and use of vasopressors were recorded. All patients were monitored using 5-lead electrocardiography, non-invasive blood pressure monitoring, and pulse oximetry. General anesthesia was induced with propofol (2 mg kg^− 1^ IV), fentanyl (3 µg kg^− 1^ IV) and rocuronium (0.6 mg kg^− 1^ IV). Anesthesia was maintained with sevoflurane (1.5–3%) and 10 mg rocuronium / 30 min to maintain adequate muscle relaxation. Mechanical ventilation commenced initially with volume-controlled ventilation mode, V_T_ = 6 ml Kg^− 1^ (PBW), Positive end-expiratory pressure (PEEP) = 3–5 cmH_2_O, respiratory rate = 10–12 breath/minute, and a fraction of inspired oxygen (FiO_2_) = 0.5–0.7 (Oxygen air mixture) to achieve and maintain a peripheral oxygen saturation (SPO_2_) ≥ 96%. The PBW (kg) was calculated as follows: X + 0.91[height (cm) − 152.4]; (X = 50 for men and = 45.5 for women). GE Avance® CS 2 anesthesia machines (*GE Healthcare, Madison, WI*, USA).

A 7 F triple-lumen central venous catheter was inserted in the right internal jugular vein with real-time ultrasound guidance and complete aseptic precaution. A pressure transducer was connected to the 16-gauge lumine to continuously monitor the central venous pressure (CVP). The left radial artery was cannulated for measuring mean arterial pressure (MAP) invasively. The pressure transducers for both CVP and invasive arterial blood pressure were placed on the midaxillary line, fixed to the operating table to keep the sensor at the atrial level, and zeroed to atmospheric pressure. An indwelling urinary bladder catheter was inserted to monitor urinary output. Head and extremity wrap and forced warming system were applied to maintain body temperature. A trans-esophageal doppler probe (*Cardio QP EDM™; Deltex Medical, Chichester*, UK) was greased with a lubricating gel and passed nasally into the mid-esophagus until the aortic blood flow signals were best identified. The Masimo Pulse Co-Oximeter probe (*Masimo SET Rainbow R2-25r and R225a; Masimo Corp., Irvine, CA*, USA) was placed on the index finger of patients and covered to avoid light interference. The Stroke volume (SV) and stroke volume variation (SVV) were measured directly by the trans-esophageal doppler probe. The stroke volume index (SVI) was calculated by dividing the value of SV by BSA (obtained by Cardio QP EDM™ monitor after providing age, sex, weight, and height of the patient). The PVI and Perfusion index (PI) values were reported by the aid of The Masimo Pulse Co-Oximeter monitor. Throughout surgery, packed red blood cells (300 ml) were transfused when the hematocrit level was < 25%. Fresh frozen plasma (200 ml) was administrated when the fibrinogen level was < 2 g dl^− 1^ or the international normalized ratio was > 2. Patients were extubated either in the operating room or postoperatively in the ICU after completion of surgical intervention.

## Intervention protocol (Fig. 1)

**Fig. 1:**
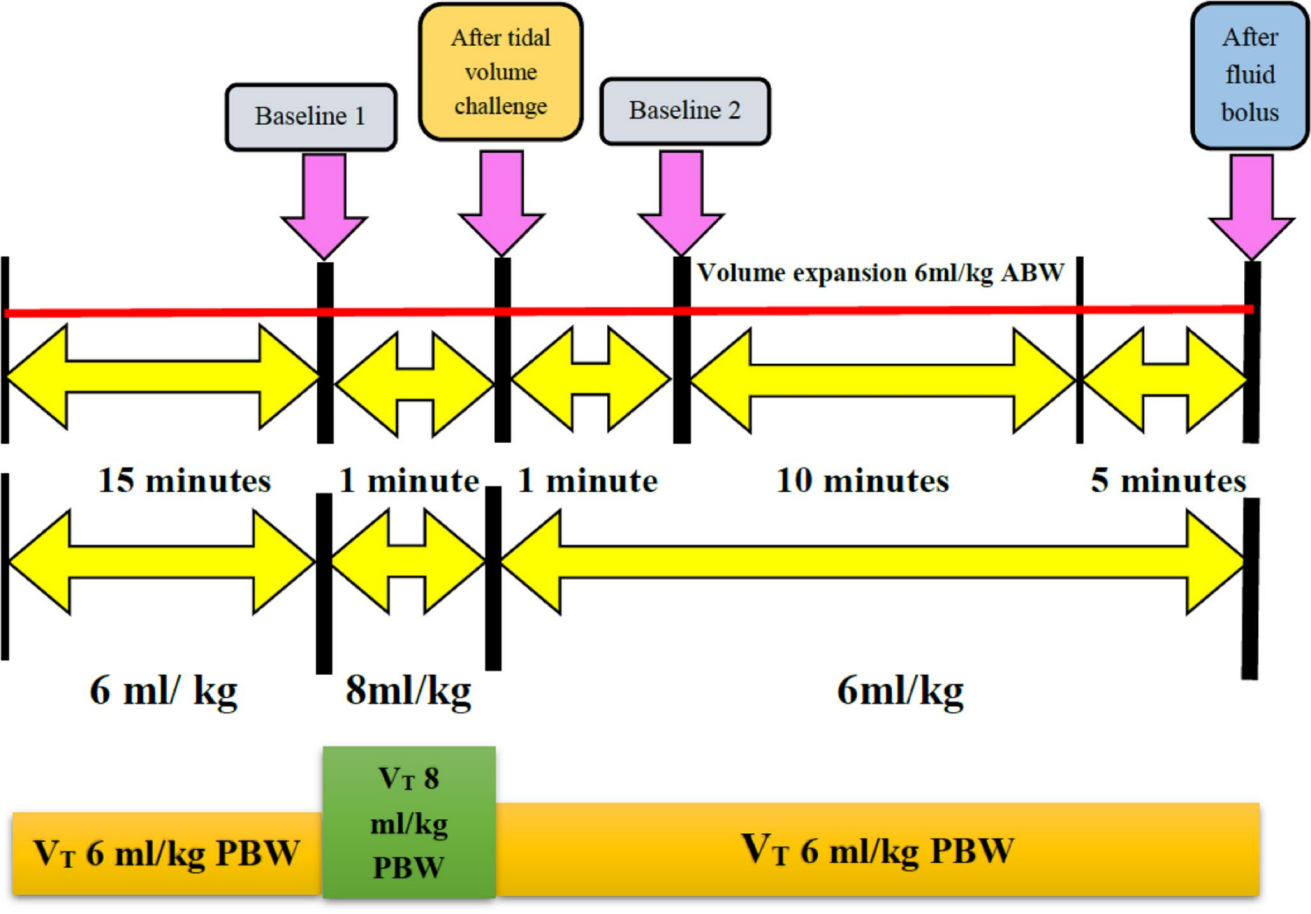
Study protocol. Arrows indicate time points at which measurements were made, V_T_: tidal volume; PBW: predicted body weight; ABW: actual body weight

After the tumor resection phase, baseline hemodynamic variables including heart rate (HR), MAP, CVP, SVI, PVI, PI, and SVV with a 6 ml kg^− 1^ V_T_ ventilation (baseline 1 time-point) were recorded. After baseline measurement, V_T_ was increased to 8 ml kg^− 1^ PBW for 1 min (after V_T_ challenge time-point), and the hemodynamic variables were obtained. The V_T_ has been reduced back to 6 ml kg^− 1^ PBW again and after 1 min, the hemodynamic measurements were recorded (baseline 2 time-point). After these hemodynamic measurements, volume expansion was performed for 10 min using an infusion of a balanced crystalloid solution (6 ml kg^− 1^ ABW). The same hemodynamic parameters were measured under ventilation with a V_T_ of 6 ml kg^− 1^ five minutes after fluid bolus administration (after fluid bolus time-point). Then, the absolute change (ΔPVI_6–8_) (PVI after − PVI before V_T_ challenge) and percentage change (%ΔPVI_6–8_) (PVI after − PVI before VT challenge / PVI before V_T_ challenge) between the PVI at 6 ml kg^− 1^ of PBW (PVI_6_) and that at 8 ml kg^− 1^ of PBW (PVI_8_) (i.e., after performing a V_T_ challenge) were calculated. The percentage change in SVI, according to volume loading, was used as the principal indicator of fluid responsiveness. Responders or non-responders were determined when the increase in SVI was ≥ 10% or < 10% after fluid bolus, respectively [14]. No more than two V_T_ challenges can be performed in any patient. Doses of vasoactive medications, if used, and PEEP were kept constant one minute before and during the four time-points of data acquisition. We defined the primary outcome as the best cut-off value of ΔPVI_6–8_ in hepatobiliary or pancreatic surgeries using a V_T_ challenge. The grey zone was defined as a range between a low cutoff value of ΔPVI_6–8_ that included 90% of fluid responders and a high cutoff value of ΔPVI_6–8_ that included 90% of fluid non-responders [15, 16].

The secondary outcomes were the evaluation of the hemodynamic variables (HR, MAP, CVP, SVI, PVI, PI, and SVV) at four time-points (V_T_ 6 ml/kg baseline 1, V_T_ 8 ml/Kg after V_T_ challenge, V_T_ 6 ml/Kg baseline 2, and after fluid bolus administration) for fluid responders and non-responders. Additionally, calculation of the area under the curve (AUC), detection of sensitivity, specificity, and best cutoff value of PVI_6_, PVI_8_, ΔPVI_6–8,_ %ΔPVI_6–8_, and other statistically significant differences of the other measured hemodynamic variables of fluid responsiveness.

## Sample size calculation and statistical analysis

The online Statstodo application (www.statstodo.com) was utilized to determine the sample size prerequisite for assessing two receiver operating characteristics (ROC) curves with anticipated AUC of 0.65 (PVI_6_) and 0.90 (ΔPVI_6–8_), presuming an *α* error = 0.05 and a power = 90%. A minimum of 40 patients were considered necessary to identify an AUC difference of 0.25 when assuming equal numbers of fluid responders and non-responders [17]. As the data loss was likely to be within 20%, 48 patients have to be enrolled in this study.

The SPSS software Version 21.0 (*IBM, Armonk, NY*, USA) was used to perform statistical analysis. Data were presented as mean ± standard deviation (SD), median [interquartile range (IQR) (range)], or percentage (%). Distribution normality was assessed using the Shapiro-Wilk test. The changes in continuous variables from 6 to 8 mg kg^− 1^ PBW (after V_T_ challenge) and the changes before and after fluid bolus were compared using the paired t-test or Wilcoxon signed rank-sum test. Group comparisons between responders and non-responders were performed using the independent t-test or Mann–Whitney U test. Categorical variables were analyzed using the chi-square test or Fisher exact test when indicated. To test the abilities of the dynamic preload indices to predict fluid responsiveness, the AUCs of responders were calculated and compared using the Hanley–McNeil test (AUC = 0.5, a useless test with no possible prediction; AUC = 0.6–0.69, a test with poor predictability; AUC = 0.7–0.79, a fair test; AUC = 0.8–0.89, a test with good predictability; AUC = 0.9–0.99, an excellent test; AUC = 1.0, a perfect test with the best possible prediction) [18]. An optimal threshold value was determined for each variable to maximize the Youden index [sensitivity + (specificity − 1)] [19].

## Results

**Fig. 2:**
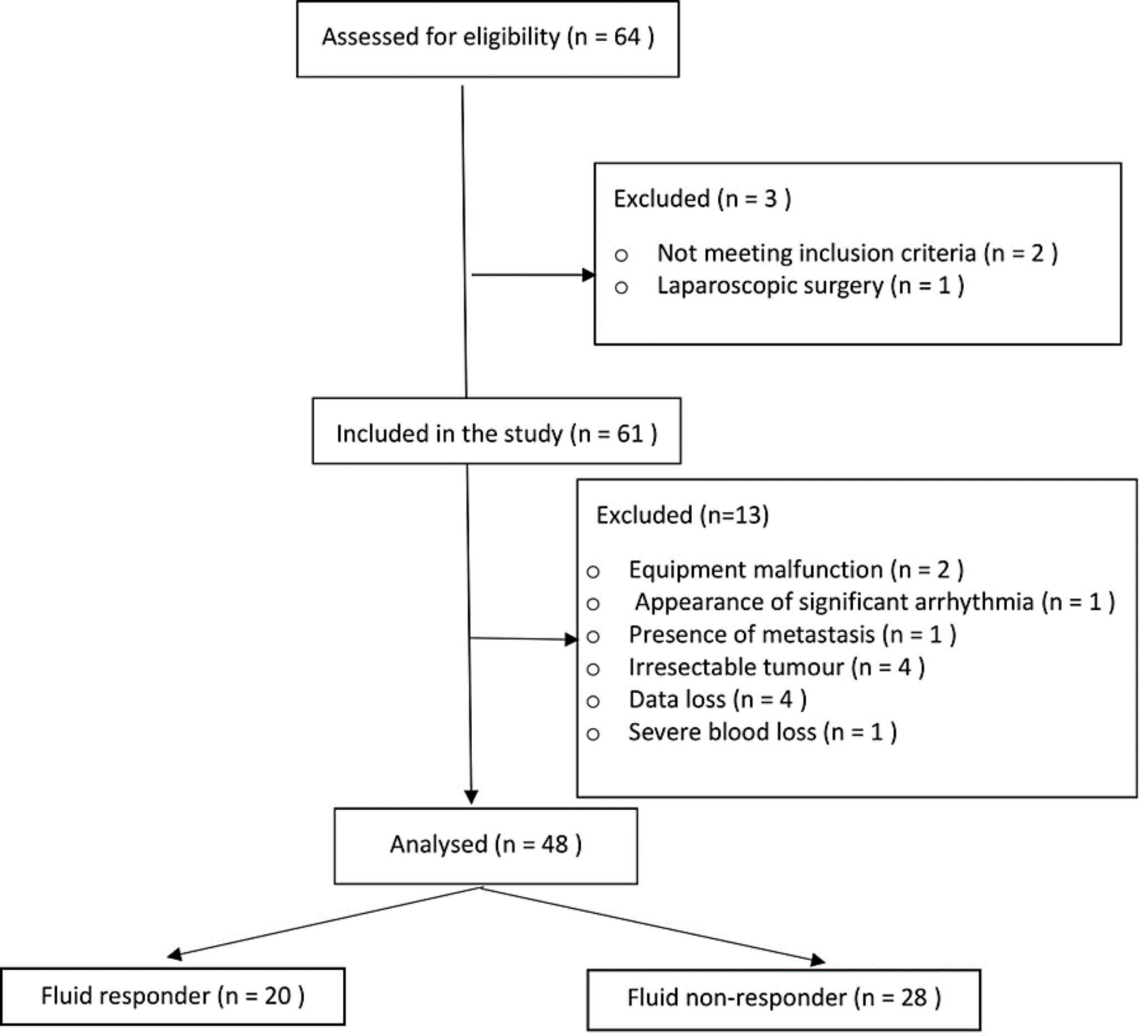
Study CONSORT flowchart diagram

**Fig. 3:**
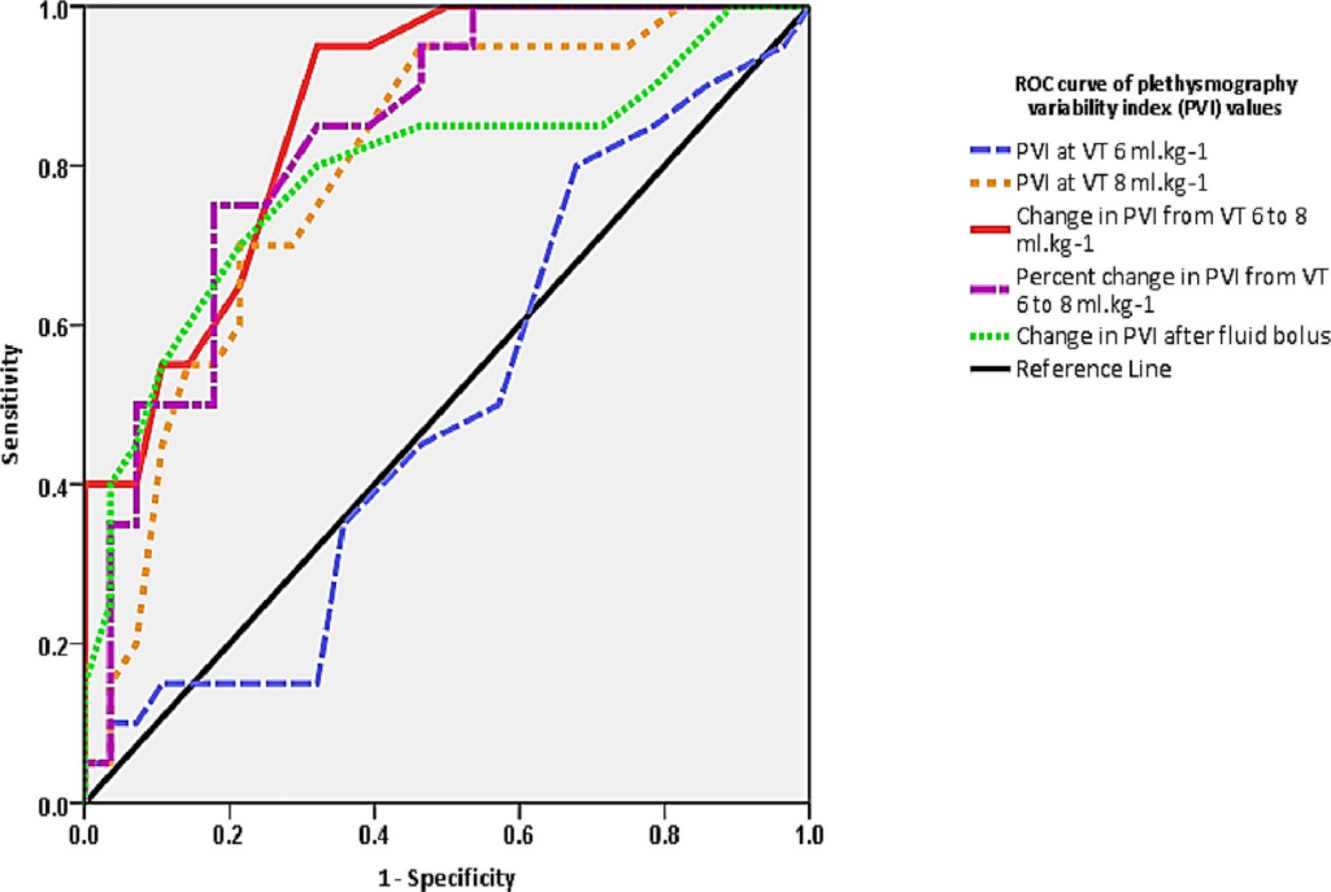
shows receiver-operating characteristic (ROC) curves of The plethysmography variability index (PVI) different parameters values for forty-eight patients undergoing tidal volume challenge

The patients in the current study were enrolled between August 2018 and February 2020. Sixty-four consecutive hepatobiliary or pancreatic tumour resection patients were considered eligible for inclusion. There were three patients excluded before and thirteen patients excluded after the enrolment. (Fig. 2) Finally, forty-eight patients were analysed, twenty patients (41.7%) were fluid responders (SVI rise by more than or equal 10% after crystalloid fluid bolus 6 ml Kg ^− 1^ ABW) while twenty-eight patients (58.3%) were fluid non-responders (SVI rise by less than 10%). Each enrolled patient experienced only V_T_ challenge once. Demographic characteristics (age, sex, weight, height, PBW, BMI, and BSA), comorbidities, surgical procedures, and vasopressors use were comparable between responders and non-responders. (Table 1) All the hemodynamic values of responders and non-responders before fluid bolus administration were comparable at Baseline 2 time-point. (Table 2) The fluid bolus administration significantly increased SVI in responders from mean ± SD 41.3 ± 9.9 ml. beat^− 1^.m^− 2^ to mean ± SD 49.6 ± 13 ml. beat^− 1^.m^− 2^, *P* < 0.001 and there was statistically significant difference between responders mean ± SD 49.6 ± 13 ml. beat^− 1^.m^− 2^ and non-responders mean ± SD 42 ± 10 ml. beat^− 1^.m^− 2^, *P* = 0.03 after fluid bolus. (Table 2) The HR and MAP values were comparable before and after the fluid bolus for responders and non- responders. The CVP values were comparable before and after the fluid bolus for fluid responders but there was statistically significant rise of CVP values for non-responders after the fluid bolus as compared to the values before the fluid bolus, *P = 0.02*. This statistical significance was of trivial clinical impact. (Table 2) There were statistically significant differences of PI values after fluid bolus for responders, *P =* 0.03 and non- responders, *P =* 0.01 but the differences were comparable between responders and non- responders before, *P =* 0.14 and after, *P =* 0.11 the fluid bolus administration. (Table 2) For SVV values, the changes were comparable between responders and non-responders before and after the fluid bolus administration. (Table 2) All the hemodynamic variables of responders and non-responders before the V_T_ challenge were comparable at Baseline 1 time-point. For the values of HR, MAP, CVP, SVI, PI, and SVV, there were no statistically significant differences between responders and non-responders after tidal volume challenge time-point. Additionally, there were no statistically significant differences of the previously mentioned variables when comparing these values before and after applying V_T_ challenge for both responders and non-responders. (Table 2) The V_T_ challenge increase PVI in fluid responders from median [IQR (range)] 9.5% [9–12 (4–29)] at V_T_ 6 ml Kg^− 1^ baseline 1 time-point to 18% [12–19 (7–35)] at V_T_ 8 ml Kg^− 1^ after V_T_ challenge, *P* < 0.001. This difference was not significant in non-responders. (Table 2) The AUC of PVI_6_ was 0.49 (95% confidence interval (CI), 0.33–0.66), *P* = 0.99 showing non-significant prediction of fluid responsiveness. The AUC of PVI_8_ was 0.79 (95% CI, 0.66–0.92), *P* = 0.001 showing a sensitivity of 85% and a specificity of 61% for a best cutoff value of 11.5%. The AUC of ΔPVI_6–8_ was 0.86 (95% CI, 0.76–0.96), *P* < 0.001 showing a sensitivity of 95% and a specificity of 68% for a best cutoff value of a 2.5%. (Table 3; Fig. 3) The ΔPVI_6–8_ values of four enrolled patients (8.3%) were within the inconclusive grey-zone range of the test (1–6%). The fluid bolus cause PVI values reduction in fluid responders from median [IQR (range)] 11.5 [9.2–16 (5–29)] at V_T_ 6 ml Kg^− 1^ baseline 2 time-point to 9 [5.5–10 (3–17)] at V_T_ 6 ml Kg^− 1^ after fluid bolus, *P* < 0.001. This difference was not significant in non-responders, *P* = 0.12. The differences between fluid responders and non-responders were comparable at the same time-points. (Table 2) The AUC of change of PVI after fluid bolus was 0.78 (95% CI, 0.65–0.92), *P* = 0.001 showing a sensitivity of 70% and a specificity of 79% with a best cutoff value of 2%. (Table 3; Fig. 3)

**Table 1:** Demographics and operative characteristics of the forty-eight included patients undergoing tidal volume challenge

Characteristic	Overall (n = 48)	Responder (n = 20)	Non-responder (n = 28)
Age (years)	53.1 (12.5)	53.9 (11.8)	52.5 (13.1)
Sex Male Female	38 (79%) 10 (21%)	17 (85%) 3 (15%)	21 (75%) 7 (25%)
Height (cm)	170.7 (5.6)	171.4 (6.2)	170.2 (5.2)
Predicted body weight (Kg)	65.6 (6)	66.6 (6.2)	64.9 (5.8)
Body mass index (Kg.m^− 2^)	26.2 (3.4)	25.4 (2.3)	26.8 (3.9)
Body surface area (m^2^)	1.9 (0.17)	1.8 (0.16)	1.9 (0.18)
Smoking history Yes No	32 (67%) 16 (33%)	16 (80%) 4 (20%)	16 (57%) 12 (43%)
Comorbid diseases No Yes Hypertension Diabetes Combined Others	29 (60%) 19 (40%) 6 (13%) 4 (8%) 4 (8%) 5 (11%)	12 (60%) 8 (40%) 3 (15%) 2 (10%) 1 (5%) 2 (10%)	17 (61%) 11 (39%) 3 (11%) 2 (6%) 3 (11%) 3 (11%)
Type of operation Hepatic tumor resection Pancreatic tumor resection Biliary tumor resection	24 (50%) 23 (48%) 1 (2%)	9 (45%) 10 (50%) 1 (5%)	15 (54%) 13 (46%) 0 (0%)
Use of vasopressors Yes No	11 (23%) 37 (77%)	4 (20%) 16 (80%)	7 (25%) 21 (75%)

Values are expressed as mean (SD) or number (proportion %)

**Table 2:** Hemodynamic variables in responders (n = 20) and non-responders (n = 28)

	Baseline 1 (Tidal volume 6 ml kg^− 1^)	After tidal volume challenge (Tidal volume 8 ml kg^− 1^)	*P* value	Baseline 2 (Tidal volume 6 ml kg^− 1^)	After fluid bolus	*P* value
HR (beats.min^− 1^) Responder Non-responder *P* value	84 (16) 90 (18) 0.28	89 (19) 91 (17) 0.67	0.06 0.29	86 (16) 89 (16) 0.53	87 (13) 91 (16) 0.35	0.66 0.07
MAP (mmHg) Responder Non-responder *P* value	83 (14) 78 (11) 0.2	83 (19) 80 (10) 0.46	0.95 0.37	81 (12) 81 (9) 0.8	83 (12) 84 (10) 0.79	0.55 0.09
CVP (cmH_2_O) Responder Non-responder *P* value	6.5 (2.5) 8 (2.5) 0.14	7.5 (3.5) 8.5 (2.5) 0.42	0.06 0.08	7.5 (3) 9 (2.5) 0.1	8 (3.5) 9.5 (2.5) 0.09	0.55 0.002^*^
SVI (ml. beat^− 1^.m^− 2^) Responder Non-responder *P* value	43 (11.2) 41.6 (9.7) 0.66	42.5 (11.2) 42 (10.2) 0.87	0.72 0.57	41.3 (9.9) 42.5 (9.5) 0.67	49.6 (13) 42 (10) 0.03^*^	< 0.001^*^ 0.52
PVI (%) Responder Non-responder *P* value	9.5 [9–12 (4–29)] 10 [8–13.7 (4–26)] 0.99	18 [12–19 (7–35)] 10 [7.2–13 (5–32)] 0.001^*^	< 0.001^*^ 0.55	11.5 [9.2–16 (5–29)] 10 [6–12 (5–20)] 0.05	9 [5.5–10 (3–17)] 8 [6.2–11.7 (3–20)] 0.95	< 0.001^*^ 0.12
PI (%) Responder Non-responder *P* value	1.5 [0.84–3.5 (0.37–6.8)] 1.4 [0.55–2.8 (0.15–4.9)] 0.32	1.6 [0.71–3 (0.48–5.9)] 0.82 [0.46–2.4 (0.12–6.3)] 0.14	0.22 0.06	1.5 [0.67–3.2 (0.27–8.4)] 0.78 [0.52–2.5 (0.13–5.4)] 0.14	1.9 [1–3.5 (0.24–9.6)] 0.97 [0.38–2.6 (0.31–7.3)] 0.11	0.03^*^ 0.01^*^
SVV (%) Responder Non-responder *P* value	13 [9–16.7 (5–26)] 11.5 [8.2–14.7 (5–23)] 0.41	12.7 [10.2–17.7 (8–21)] 11.5 [8–17.5 (7–21)] 0.22	0.97 0.69	11.9 [10.2–14.2 (6–25)] 11 [9–13.7 (5–20)] 0.4	12.5 [9.7–14 (6–28)] 11.5 [8–14.7 (6–21)] 0.4	0.3 0.57

Values are expressed as mean (SD) or median [IQR (range)]

HR: heart rate; MAP: mean arterial pressure; CVP: central venous pressure; SVI: Stroke volume index; PVI: plethysmography variability index; PI: perfusion index; SVV: stroke volume variation. * = P < 0.05

**Table 3:** Diagnostic ability of various variables to predict fluid responsiveness in the included forty-eight patients undergoing tidal volume challenge with the best cut-off, sensitivity, and specificity values for significant variables

	Area Under the Receiver-Operating Characteristic Curve (95% CI)	*P* value	Best Cut-off Value	Sensitivity (%)	Specificity (%)
HR at V_T_ 6 ml/kg PBW	0.41 (0.25–0.57)	0.32	-	-	-
HR at V_T_ 8 ml/kg PBW	0.44 (0.27–0.61)	0.54	-	-	-
MAP at V_T_ 6 ml/kg PBW	0.57 (0.40–0.74)	0.38	-	-	-
MAP at V_T_ 8 ml/kg PBW	0.53 (0.36–0.71)	0.65	-	-	-
CVP at V_T_ 6 ml/kg PBW	0.37 (0.20–0.53)	0.13	-	-	-
CVP at V_T_ 8 ml/kg PBW	0.41 (0.24–0.58)	0.30	-	-	-
SVI at V_T_ 6 ml/kg PBW	0.54 (0.37–0.71)	0.63	-	-	-
SVI at V_T_ 8 ml/kg PBW	0.53 (0.37–0.70)	0.66	-	-	-
PVI at V_T_ 6 ml/kg PBW	0.49 (0.33–0.66)	0.99	-	-	-
PVI at V_T_ 8 ml/kg PBW	0.79 (0.66–0.92)	0.001^*^	11.5%	85	61
Change in PVI from V_T_ 6 to 8 ml/kg PBW	0.86 (0.76–0.96)	< 0.001^*^	2.5%	95	68
Percentage change in PVI from V_T_ 6 to 8 ml/kg PBW	0.83 (0.72–0.94)	< 0.001^*^	29%	82	75
Change in PVI after fluid bolus	0.78 (0.65–0.92)	0.001^*^	2%	70	79
PI at V_T_ 6 ml/kg PBW	0.58 (0.42–0.74)	0.32	-	-	-
PI at V_T_ 8 ml/kg PBW	0.62 (0.46–0.78)	0.14	-	-	-
SVV at V_T_ 6 ml/kg PBW	0.57 (0.40–0.73)	0.41	-	-	-
SVV at V_T_ 8 ml/kg PBW	0.60 (0.44–0.76)	0.22	-	-	-

HR: heart rate; V_T_: tidal volume; PBW: predicted body weight; MAP: mean arterial pressure; CVP: central venous pressure,

SVI: Stroke volume index; PVI: plethysmography variability index; PI: perfusion index; SVV: stroke volume variation

* = P < 0.05, Dashes indicate that the variable was not measured as P ≥ 0.05

## Discussion

This study reveals the ability of V_T_ challenge to enhance the PVI potential to predict fluid responsiveness for non-laparoscopic hepatobiliary or pancreatic tumors resection procedures in mechanically ventilated patients under general anesthesia. The absolute change in PVI (ΔPVI_6–8_) reliably predicts fluid responsiveness with a cutoff value of 2.5% with excellent prediction of most of fluid responders, 95% sensitivity and with uncertain prediction of fluid non-responders (could miss one in every three fluid non-responders) (30% of non-responders recognized imperfectly as fluid responders), specificity 68%. The PVI_6_ has no potential to predict fluid responsiveness efficiently and the inconclusive grey-zone range of ΔPVI_6–8_ lies between 1% and 6% indicate uncertain predictive value. It is a quite wide range, but the total number of patients involved within the inconclusive grey-zone was small. There is associated intrathoracic pressure rise with providing mechanical ventilation using higher V_T_ of 8 ml kg^− 1^. The subsequent cardiopulmonary interactions significantly lower the cardiac preload status in certain groups of patients (fluid responders). This effect is masked when using a low V_T_ ventilation that minimizes these cardiopulmonary interactions and makes it difficult to differentiate between the fluid responders and non-responders. Transient increase of V_T_ during mechanical ventilation uncover this effect and help to identify the fluid responders [11]. The inherit limitations of PVI reliability of prediction of fluid responsiveness for spontaneously breathing patients, marked arrythmias, increased intraabdominal pressure [20, 21] decreased lung compliance, and right ventricular dysfunctions [21–24] might be attributed to dysregulated respiratory alterations and cardiopulmonary interactions in such conditions. Cannesson and colleagues concluded that PVI predicts fluid responsiveness efficiently in a group of patients under mechanical ventilation after anesthesia induction for coronary artery bypass grafting. They used V_T_ 8–10 ml Kg^− 1^ continually throughout data acquisition time and they found PVI was reduced from 14 to 9% after 500 ml hetastarch 6% fluid bolus. They determined a cutoff value of PVI = 14% for fluid responsiveness with 81% sensitivity and 100% specificity. They highlighted a significant relationship between PVI values change and cardiac index variations after the fluid bolus. They waited equilibrium for four minutes (3 min after volume expansion and one minute after no stimulation) after fluid bolus to collect the hemodynamic variables to assess the effects of volume expansion on cardiac output and other hemodynamic variables [22]. The findings of the current trial are consistent with that of Desebbe and colleagues. They showed a substantial differences of PVI values when using V_T_ ≥ 8 ml kg^− 1^ as compared with 6 ml Kg^− 1^ in prediction of hemodynamic instability before applying sudden positive PEEP value of 10 cmH_2_O with acceptable sensitivity and specificity in a group of patients who were sedated and mechanically ventilated postoperatively [25]. They demonstrated that differences of PVI values obtained during lower V_T_ ventilation (6 ml kg^− 1^) could not predict the hemodynamic instability efficiently following sudden PEEP application [25]. In the same context, Desgranges and colleagues used 8 ml Kg^− 1^ V_T_ for mechanical ventilation after anesthesia induction to compare three pulse oximeter sensors sites (the finger, ear, and forehead). They reported that the three sensor sites were able to predict the fluid responsiveness efficiently and comparably between the three sites [26]. The V_T_ challenge during mechanical ventilation under anesthesia was used efficiently to improve the capabilities of the dynamic indices for fluid responsiveness prediction [12, 13]. Messina and colleagues applied tidal volume challenge in a group of neurosurgical patients in prone position. They reported that the V_T_ challenge using 8 ml Kg^− 1^ V_T_ could increase the predictability of the pulse pressure variation (PPV) and SVV for fluid responsiveness with favorable sensitivity and specificity to avoid unnecessary intravenous fluid administration of such critical group of patients [13]. Their findings were emphasized on other cohort of elective neurosurgical patient on supine position [12]. In contrast to the findings of Messina and colleagues, we found that V_T_ challenge could not improve the reliability of SVV to predict fluid responsiveness as there were insignificant differences between the responders and non-responders at all time-points of assessment in addition to that all comparisons before, during, and after V_T_ challenge were comparable. This discrepancy could be attributed to the different methods of measurement of SVV or different cohort of patients between the current trial and that trials of Messina and colleagues [12, 13]. In contrast to the findings of the current trial, Myatra and colleagues reported significant difference of SVV values after V_T_ challenge 8 ml Kg^− 1^ with sensitivity 75% and specificity 76% in a group of ICU patients who were sedated, mechanically ventilated, diagnosed with septic shock, and with possibly defective lung compliance. They obtained SVV values by transpulmonary thermodilution method [11]. The values of HR and MAP were comparable before and after V_T_ challenge, before and after fluid bolus, and between fluid responders and non-responders at all time points of assessment. These findings emphasized the poor ability of these hemodynamic variables to differentiate between fluid responders and non-responders. There was statistically significant difference for CVP values before and after the fluid bolus for the fluid non-responders only but of minute clinical effect. This difference may highlight that an increase in preload in fluid non-responders will not augment cardiac output and could impair the cardiac function by increasing the preload of right ventricle. CVP values before and after V_T_ challenge for both fluid responders and non-responders were comparable and the CVP values before and after fluid bolus for responders were comparable denoting the inability of this parameter to predict fluid responsiveness efficiently. The current study utilized the independent SVI variables as a cardiac output surrogate to differentiate between fluid responders and non-responders. It is calculated by dividing the SV value (obtained by trans-esophageal doppler probe) by BSA. It is not as accurate as the values obtained by the more invasive pulmonary artery catheter with standard thermodilution method [22]. but probably it is more reliable than the non-calibrated peripheral pulse contour analysis values of SV [11]. or values determined by the non-invasive bioreactance methods [27]. The minimally invasive trans-esophageal doppler measure the stroke distance (flow velocity multiplied by flow time) of the descending part of thoracic aorta. SV is calculated as Stroke distance multiplied by aortic cross-section area (which is an area of a circle π*r*^2^). The radius of the descending aorta (which is used to calculate aortic cross-section area) is derived from published nomograms based on age, sex, weight, and height (*Deltex, West Sussex*, England, www.deltexmedical.com) [28]. The resulting values of SV were then compensated (by 30%) for the fraction of blood flow that circumvented to coronaries and aortic arch branches (cerebral and upper limbs blood flow) [28–30]. The 10% cutoff value to differentiate fluid responders and non-responders was utilized before for the same aim by Messina and colleagues who obtained the SV values by radial artery waveform analysis [12]. The values of PI before and after the V_T_ challenge for both fluid responders and non-responders were comparable. On the other hand, there were statistically significant differences of PI values before and after fluid bolus for both the fluid responders, *P* = 0.03 and non-responders, *P* = 0.01. This effect could be interpreted as PI is not a good predictor of fluid responsiveness, but fluid bolus administration could increase the values of PI in the fluid responders and non-responders similarly without consequent impact on the cardiac output. The PI value changes may possibly suggest intravascular volume status changes that not based on respiratory alterations and cardiopulmonary interactions. These findings go in line with the findings of Cannesson and colleagues [22].

## Study limitations

The current trial is a non-randomized study recruiting 64 successive patients for hepatobiliary and pancreatic tumor resection. Its findings should be manipulated and interpreted carefully as data acquisition done under rigorous controlled circumstances in selected group of patients. We did unplanned additional patients’ exclusion before and after enrolment process to keep this non-randomized cohort of patients homogenous under same standard circumstances to minimize the risk of bias. Before enrollment, one laparoscopic hepatobiliary tumor resection case was excluded as the pneumoperitoneum could increase intraabdominal pressure with subsequent affection of respiratory alteration and heart-lung interactions that could interfere with PVI values changes and accuracy of fluid responsiveness prediction. Tissue dissection, third space fluid losses, fluid shifts, and intraoperative blood loss were supposed to be less in laparoscopic surgery than in open type procedures. An additional factor to exclude this case is frequent position changing (head up and down positions). There were four cases of irresectable tumors, and one case of undiagnosed metastasis that were excluded from the study after enrolment as these inoperable cases were subjected to less negligible tissue dissection, fluid shifts, and blood loss than the patients undergone complete tumor resection who may require more volume expansion, blood transfusion, or vasoactive drugs administrations. There was one case of massive blood loss excluded after enrollment as the patient needed massive blood transfusion with high dose vasopressors and experienced marked hemodynamic instability that precluded protocol completion. The five minutes interval between completion of crystalloid fluid bolus (over ten minutes) and acquisition of hemodynamic variable after fluid bolus was relatively long. This delay might result in fading and possibly underestimating the assumed clinical effects of volume expansion and the final outcome.

## Conclusion

For optimizing intraoperative fluid administration in hepatobiliary and pancreatic surgeries, V_T_ challenge improves the prediction accuracy of PVI to reliably identify fluid responders and non-responders as changes in PVI values obtained by transient increase of V_T_ to 8 ml Kg^− 1^ are more predictive than PVI values that are reported during low tidal volume ventilation (6 ml Kg^− 1^).

## Glossary

ABW: Actual body weight.
AUC: area under the ROC curve.
CI: cardiac index.
CVP: central venous pressure.
FiO2: fraction of inspired oxygen.
HR: heart rate.
ICU: intensive care unit.
IQR: interquartile range.
MAP: mean arterial pressure.
PBW: predicted body weight.
PEEP: Positive end-expiratory pressure.
PPV: pulse pressure variation.
ROC: receiver operating characteristic.
SD: standard deviation.
SPO_2_: Peripheral oxygen saturation.
SV: stroke volume.
SVI: stroke volume index.
SVV: stroke volume variation.
V_T_: tidal volume.
